# Drought Stress Tolerance in Plants

**DOI:** 10.3390/ijms24076562

**Published:** 2023-03-31

**Authors:** Esther M. González

**Affiliations:** Institute for Multidisciplinary Research in Applied Biology (IMAB), Public University of Navarra, 31006 Pamplona, Spain; esther.gonzalez@unavarra.es

The current climate change scenario is accelerating degradation, desertification, and salinisation: all destructive processes that are negatively impacting arable lands and food production. This is particularly important when considering how the world population shows a marked positive trend. This scenario leads to flooding and decreasing water quality, but also to a decrease in the availability of water resources in some regions. More than ever, drought is a significant threat to agriculture worldwide. This Special Issue focuses on recent advances in the mechanisms involved in the drought tolerance of crop plants, with particular attention to the role of the root tissue and shoot–root interactions. In addition to drought, it considers other abiotic stresses involving water deficit stress at the cell level and their interactions with drought. The Special Issue includes a review paper and a collection of scientific papers that approach drought stress in cereals, legumes, and trees, combining studies in cultivated, wild, and model plants. Overall, this issue remarks the role of transcriptions factors (*bHLH, NAC, HD-ZIP III*), leucine-rich repeat receptor-like kinases, cytochrome P450 monooxygenases, and U-box E3 ligases in drought stress responses at different levels. In addition, the interaction between plant nutrition and drought stress responses is approached with a physiological strategy.

Regarding cereals, Panda et al. [1] reviewed the critical traits of root system architectures (RSA) to increase resource-use efficiency and grain yield in rice direct-seeded under aerobic soil conditions. Direct-seeding (DSR) is a water-saving alternative to conventional puddled transplanted rice, and RSA traits are the major component in the DSR ecosystem to boost productivity. The advances in molecular breeding technologies, TILLING approaches, and the MAS strategy provide an opportunity to identify key RSA traits in the DSR ecosystem. Candidate genes such as early root growth enhancers, surface rooting QTL, deep rooting genes, numerous nutrient transporters, and stress-responsive factors have been shown to play a role in stress adaptation. These new high-throughput root phenotyping platforms provides a new step toward filling the gap between field and laboratory analysis achieving a deeper understanding of specific root traits and opening new horizons to the design of an ideal drought-tolerant root architecture. Sehar et al. [2] studied drought stress tolerance in cultivated and wild Tibetan barley and its interactions with potassium nutrition. Overall, water and potassium limitations attenuated plant growth, but the tolerant wild barley cultivar was least affected due to its ability to retain potassium in its tissues which exhibited smaller reductions in photosynthetic parameters and the lowest Na+/K+ ratios. Therefore, Tibetan wild barley genotypes that utilise potassium efficiently could serve as a valuable genetic resource for improved potassium metabolism and better tolerance for abiotic stresses. Kim et al. [3] isolated and characterised two wheat U-box E3 ubiquitin ligase genes (TaPUB2 and TaPUB3) that were highly expressed in response to abiotic stresses. These proteins target protein intramolecular interactions for scaffold stabilisation. Their expression patterns, subcellular localisation, and activity were elucidated in this study, demonstrating how the two proteins directly interact with one another and form heterodimeric complexes in the cytoplasm of wheat protoplasts. The heterogeneous overexpression of both genes in *Arabidopsis* conferred tolerance to drought stress supporting the heterodimeric form of U-box E3 ubiquitin ligases’ role as a positive regulator of drought stress tolerance.

Regarding legumes, Xia et al. [4] focused on identifying cytochrome P450 monooxygenases that responded to drought/salt stress in the model legume *Medicago truncatula*. In this study, 346 *MtP450* genes were identified and classified into ten clans containing 48 families. The expression pattern of 204 *Mt450* genes was analysed, and fourteen genes were categorised as hub genes under drought or NaCl treatments. These hub genes, as induced by stress treatments, were involved in secondary metabolism and fatty acid pathways, including scavenging ROS, promoting stomatal closure, and affecting plant development and hormone homeostasis. Identifying these *MtP450* genes provided new clues for function characterisations and their possible application in the stress-related breeding of *Medicago* and legume crops. Li et al. [5] studied the regulation of auxin biosynthesis under drought stress in *Caragana korshinskii*, a shrub native to desert areas that are highly tolerant to salty, cold, and dry conditions. The transcription factor, *C. korshinskii REVOLUTA*(*CkREV*), can bidirectionally regulate the expression of the critical enzyme gene *CkYUC5* in auxin synthesis according to external environmental changes, to control the biosynthesis of auxin and further enhance the drought resistance of plants. CkREV can positively regulate the expression of *CkYUC5* to promote auxin synthesis in favour of growth under normal development. However, *CkREV* can also respond to external signals and negatively regulate the expression of *CkYUC5*, which inhibits auxin synthesis to reduce the growth rate, lower water demands, and eventually improve the drought resistance of plants.

Regarding tree crops, poplar is one of the most important tree species in the north temperate zone with high water requirements. Li et al. [6] showed that the overexpression of the *PdERECTA* gene, a member of the *LRR-RLKs* (Leucine-rich repeat receptor-like kinases) family, improves water use efficiency and enhanced drought tolerance by reducing stomatal density. Overexpressed *PdERECTA* in plants caused them to survive longer after watering stopped, exhibiting better photosynthesis conditions, a faster growth rate, and higher biomass accumulation, thus making this gene a promising candidate for the improvement of trees’ drought tolerance. On the other hand, the Special Issue includes two papers about *Hibiscus*: a pioneer tree species that is extremely resistant to salt and drought which integrates medicinal, edible, health care, ornamental, greening, water, and soil protection, together with fibrous raw material values. Ni et al. [7] explored the expression of the basic helix-loop-helix (*bHLH*) transcription factors, and Wang et al. [8] explored the family of *NAC* transcription factors in *Hibiscus* regarding its role in abiotic stress responses and increasing salt tolerance. The overexpression of *HhbHLH2* or *HhNAC54* in *Arabidopsis thaliana* significantly increased tolerance to salt by modulating the root architecture. These results provide a basis for a comprehensive analysis of *bHLH* and *NAC* transcription factor families and insights into the abiotic stress response mechanisms.

Summarising, this Special Issue studies a representative collection of species integrating the knowledge from model species with that of economically important crops. Different transcription factors, together with hormone metabolism regulators and nutrient status, play a critical role in plant drought stress, contributing to a better understanding of the mechanisms that are implicated as routes to improving drought tolerance in crop species.
